# Getting serious about the challenge of regulating germline gene therapy

**DOI:** 10.1371/journal.pbio.3000223

**Published:** 2019-04-30

**Authors:** Arthur Caplan

**Affiliations:** Division of Medical Ethics, New York University School of Medicine, New York, New York, United States of America

## Abstract

The announcement of He Jiankui’s germline editing of human embryos has been followed by a torrent of almost universal criticism of the claim on scientific and ethical grounds. That criticism is warranted. There is little room for anything other than vociferous condemnation of He's announcement. Presenting the results of groundbreaking work by press conference and YouTube is not science. The issue now is not whether the work supporting the claims reported from China was done in an ethical manner. It was not. What is required to move forward is a justification for doing germline editing in humans. Many think there is none, and prohibitions abound. If such work is justifiable, a serious, rigorous framework must be imposed that insures that such research is done following the highest ethical standards that both protect human subjects and insure public trust and support.

## Abstract 2

The announcement of an experiment on human embryos using germline genetic engineering has been met with near universal condemnation on ethical and scientific grounds. But should germline genetic research still be pursued, and if so, why? This Perspective article argues that it should, but only if it is conducted under a transparent and enforceable ethical/regulatory framework

## Appropriate outrage over the first claimed germline experiment in human embryos

He Jiankui, a scientist at the Southern University of Science and Technology in Shenzhen, China, announced November 25th that he had created the first babies from germline-edited human embryos. At a rapidly convened session during the Second International Summit on Human Genome Editing in Hong Kong, Jiankui claimed that twin girls, Lulu and Nana, were doing well and at home with their mother, Grace, and father, Mark. “Grace started her pregnancy by regular IVF (in vitro fertilization) with one difference,” Jiankui said. “Right after we sent her husband’s sperm into her eggs, we also sent in a little bit of protein and instructions for a gene surgery.” The surgery, Jiankui explained, “removed the doorway through which HIV enters to infect people.” More genetically altered children, Jiankui stated, were on the way [1].

Jiankui said his aim was prevention—to make it harder for HIV to infect the babies by creating enhanced resistance in them. The girls, he stated, needed this protection because their father carried the HIV virus. That rationale was immediately challenged by many experts on preventing HIV transmission from fathers to children who noted that existing techniques are already available and do not require gene editing, thereby undermining the stated rationale for the experiment [2].

Since this announcement, there has been almost universal criticism of the claim of germline gene editing in humans on scientific and ethical grounds. China’s government ordered a halt to Jiankui's work. The Chinese Vice Minister of Science and Technology labeled the research illegal and unacceptable. A group of more than 100 prominent Chinese scientists [3] declared Jiankui's work outside the boundaries of acceptable science [4]. The Chinese government has drafted rules including fines and bans on doing research for any unauthorized experiments [5]. Professor Jiankui has barely been seen or heard from since his appearance at the November meeting. There is little room for anything other than vociferous condemnation of Jiankui's announcement. A deep understanding of the mechanisms and potential side effects of embryo editing is an absolute prerequisite to any further discussion on its implementation. At present, human embryonic editing, particularly in regard to how DNA is repaired following introduction of a CRISPR associated protein 9 (Cas9)-induced break, is poorly understood [6].

Presenting the results of groundbreaking work by press conference and YouTube is not science. Although a paper was submitted by Jiankui and his team to at least 1 peer-reviewed journal, announcing a groundbreaking experiment prior to publication leaves no room for adequate assessment of either the science or the ethics involved.

It is also unclear whether the media, particularly the Associated Press and MIT Technology Review, where the experiment was first reported, had advanced knowledge of Jiankui's work due to a prior agreement with him for an exclusive. Showing more concern about press coverage than disseminating details of the work in the peer-reviewed literature is culpable grandstanding.

Conflict of interest also appears to have tainted this first use of gene editing. Patents were being sought [7], and clients were attracted to the infertility program where Jiankui worked and where he and his team stood to profit by recruiting subjects to the clinic with promises of disease prevention.

The consent form given to the parents was utterly inadequate. It was 23 complex pages but spent more time on who controlled baby pictures than on risks. And there appears to have been relatively little effort made to ensure parental comprehension [8].

But the issue now is not whether the work supporting the claims reported from China was done in an ethical manner. It was not [2]. What is required to move forward is a justification for doing germline editing in humans. Many think there is no justification, and prohibitions abound [9,10,11,12]. If such work is justifiable, a serious framework must be imposed that insures that the research is done following the highest ethical standards that both protect human subjects and insure public trust and support [13].

## Justifying the goal of germline editing

Some critics of germline gene editing in humans contend that there is no basis for pursuing it. They note that most of the genetic diseases for which germline editing is proposed as therapy can be avoided by preimplantation genetic testing of embryos and disposal of those showing defects or risk factors. Beyond some very rare diseases, the only purpose for using germline editing in humans, critics contend, is for enhancement—to improve the qualities and traits of offspring. But the critics of the goals of germline interventions are wrong.

Screening embryos is useful, but it does not eliminate disease forever. And offering Preimplantation genetic testing (PGD) and embryo disposal is not an option that all parents find either morally or economically acceptable. Germline editing holds out the promise of eliminating various genetic scourges from families and, ultimately, the human species for all time. Not all forms of enhancement are prima facie wrong. Bestowing improved immunity or disease resistance on future offspring seems noble not unethical. Effective and safe germline editing has a place in medicine. The fact that not every possible genetic alteration is morally defensible does not mean that none are.

## Gene editing—A regrettable history of dubious ethical conduct

Another reason to insist upon the creation of a serious ethical framework for guiding germline research is the history of dubious behavior with respect to pioneering gene editing. Many of the early pioneers of gene editing, including Stanfield Rogers, who engaged in a very early viral vector study that was very poorly understood, and Martin Cline of the University of California at Los Angeles (UCLA) School of Medicine, who attempted to perform gene therapy to transfect the beta-globin gene into autologous bone marrow cells in two patients with thalassemia, one in Israel and one in Italy violated core tenets of research ethics. Cline's protocol had been disapproved by the Institutional Review Board (IRB) and lost all federal research funding. When accused of jumping the gun, much as Jiankui has now been, Cline responded that he was uniquely qualified to try the experiment,

- “I say the answer is not. I realize that I was taking the risk of drawing criticism for such experiments. But I don’t know of anyone in the country who has precisely the same type of skills that I have, with knowledge both in the animal systems and in clinical investigations in man. I think that in that sense, I must be unique. In the last analysis one must ask how responsible an investigator has been up to that point in time” [14, p28].

James Wilson and his group at Penn saw a subject, Jesse Gelsinger, die in an early effort to stave off the lethal impact in newborns of a metabolic disorder. Regulators banned Wilson from further clinical research and excoriated him for having a financial conflict of interest while serving as a principle investigator (PI) [15]. W. French Anderson, often referred to as the “father” of gene therapy was embroiled in controversy for what many thought was a premature effort to treat Adenosine deaminase deficiency (ADA). He, too, was found to have financial conflicts of interest (COIs) [16]. Concern was sufficient about the control of gene modification work that the National Institutes of Health (NIH) expanded a special committee, the recombinant DNA advisory committee (RAC) in 1980 to ensure public oversight of somatic cell gene editing [17].

Current polls show continuing support for gene editing efforts aimed at curing or repairing disease including germline gene editing [18]. And there is some sentiment to allow germline work to proceed without any oversight or meddling by nonscientists [19,20]. But history and the current fiasco involving unverified claims of safe germline editing for prevention achievable by other means show that national and international policies of laissez faire are absurd in terms of guaranteeing sound and ethical science, adequate subject protection, and continued public support.

## So what is needed to proceed responsibly with germline editing?

Skepticism abounds when it comes to conversation about the regulation of research in the realm of human genetics. Many note that private money could be used to sponsor efforts at germline editing humans without any regard for ethics or public opinion. However, such dismissive attitudes do not reflect an understanding of what drives researcher involvement with cutting edge genetic work. For germline editing to proceed in the wake of the fiasco of Jiankui’s Hong Kong announcement, it will take serious action, not rhetoric, to insure that accountable norms and values undergird future work. This includes the following:

1. Disclosure of all COI including with media organizations at every talk and in every publication.
2. Explicit Management of COI by institutions where work is done. Institutions, not journals, must be accountable for managing COI.
3. Media forswearing exclusives, paid and unpaid with scientists. Exclusives have no place in disseminating transformative research such as germline investigations.
4. Sophisticated informed consent including the use of quizzes for showing subject comprehension along with the use of independent subject advocates.
5. Involvement of qualified, properly trained, and composed IRBs and/or research ethics committees.
6. No publication without depositing of COI, consent documents, IRB review, methods, data, and followup plan on a public data base registry.
7. No presentations at scientific meetings without prior publication or public posting of methods and data and ethics compliance.
8. Boycotts of any nation's germline work not conforming to ethical standards at meetings and conferences.
9. Loss of access to government or taxpayer supported charity funding for ethical violations.
10. No citation by name of investigators or their institution in peer-reviewed literature of any germline work done out of compliance.
11. National and international statements outlining appropriate goals for germline gene editing based on public hearings and/or input.
12. Periodic mandatory training in ethical requirements for germline work of all post-docs and other investigators.

There may well be further elements to add to this proposed ethical infrastructure. And some of the above need much more debate and clarification. But that said, it is not sufficient to establish that germline research ought to proceed despite ethical lapses. Regulation and penalties need sufficient bite to assure the public that renegade science has no future in designing our descendants.

## Abbreviations

ADA: Adenosine deaminase deficiency
Cas9: CRISPR associated protein 9
COI: conflict of interest
IRB: Institutional Review Board
NIH: National Institutes of Health
PGD: Preimplantation genetic diagnosis
PI: Principle Investigator
RAC: recombinant DNA advisory committee
UCLA: University of California at Los Angeles
